# Impact of the Social Marketing-Based Intervention on Preconception Healthy Behaviors of Women With Sickle Cell Disease

**DOI:** 10.7759/cureus.49455

**Published:** 2023-11-26

**Authors:** Asiyeh Pormehr-Yabandeh, Teamur Aghamolaei, Zahra Hosseini, Nasibeh Roozbeh, Amin Ghanbarnezhad

**Affiliations:** 1 Social Determinants in Health Promotion Research Center, Hormozgan Health Institute, Hormozgan University of Medical Sciences, Bandar Abbas, IRN; 2 Mother and Child Welfare Research Center, Hormozgan University of Medical Sciences, Bandar Abbas, IRN

**Keywords:** preconception care, health behavior, health belief model, social marketing, sickle cell

## Abstract

Introduction: A suggested method to improve the outcomes of pregnancy with sickle cell disease (SCD) is to engage more women at reproductive age in preconception healthy behavior (PCHB). Social marketing can be a suitable strategy to achieve this goal. We aimed to assess the impact of the social marketing-based intervention on women's engagement in PCHB.

Methods: A quasi-experimental study was conducted in Bandar Abbas and Minab (the two largest cities of Hormozgan province in Iran with a high prevalence of SCD) from 2021 to 2022. A social marketing-based intervention with the main components (participation in PCHB as a "product," social media and traditional promotional channels as a "promotion," selecting healthcare centers as a "place" for implementing the intervention, and free access to medical experts and speciality as a "price") was designed based on formative research. The sample size was estimated at 140 participants in each intervention and control group. The study's main outcome was the level of engagement in PCHB, which was assessed according to the health belief model (HBM).

Results: We compared the PCHB scores of both groups. Employing healthy behavior was more dominant in the intervention group. Awareness, perceived severity, perceived susceptibility, perceived benefits, and self-efficacy increased in both groups following intervention, while perceived barriers decreased. An analysis of covariance (ANCOVA) was performed to control and moderate the effect of scores in the pretest. As observed between the adjusted averages, awareness, perceived susceptibility, perceived severity, perceived benefits, perceived barriers, self-efficacy, and engagement in the PCHB were significantly different between the control and intervention groups after the intervention. A multivariate linear regression analysis showed that awareness, perceived susceptibility, and self-efficacy were strong predictors of PCHB.

Conclusions: Social marketing-based intervention successfully increased PCHB among women of reproductive age with SCD.

## Introduction

Pregnancy with sickle cell disease (SCD) can be hazardous because the prospective mother and the newborn are at increased risk of complications [1]. Pregnancy-related physiological changes, such as increased metabolic demand, increased blood viscosity, and hypercoagulability, are exacerbated in SCD patients, increasing the risk of complications, such as a vaso-occlusive crisis, acute chest syndrome, osteonecrosis, hepatic necrosis, leg ulcers, and thromboembolic events [2]. Preconception care, i.e., services provided to women of reproductive age planning for pregnancy, is an essential component of basic primary healthcare, providing many services that can prevent, detect, and treat risk factors early before pregnancy [3].

There is increasing evidence that exposure to unhealthy lifestyle behaviors and social risk factors before or during pregnancy affect the future health of mothers, their children, and future generations [4]. Improving lifestyle behaviors and early identification of risk factors through health promotion activities (education, counselling, and public and social health assessment) are elements of good preconception care [5]. While the effectiveness of preconception care has been widely demonstrated [6], preconception care participation and structural embedding in healthcare settings remain insufficient. The decrease in involvement can be attributed to several factors. However, there are two significant reasons for this: First, pregnancy is not always planned, and second, the difficulty and ongoing effort to reach the target population and encourage them to participate more [7].

A suggested method to improve this situation is to use a customer-oriented approach, and social marketing can be a suitable strategy to achieve this goal [8]. Several studies have been conducted to implement preconception care programs to involve more women in preconception healthy behavior (PCHB), and some have successfully led to increased awareness and subsequent participation [9,10]. However, these studies were less client-oriented and not designed with a social marketing base. Social marketing involves identifying a compelling "marketing mix" of product, price, promotion, and place to offer an exchange with clear and effective benefits, minimal barriers, and an advantage over the competition [11]. Health behavior theories can often be used to shape the development of new products or services and evaluate their effectiveness in achieving intended outcomes [10]. In the current study, we used the health belief model (HBM) to assess the efficacy of the intervention. According to the importance of the topic, this study was conducted with the general aim of investigating the impact of social marketing-based intervention on women's engagement in PCHB.

## Materials and methods

We aimed to test the hypothesis that a multicomponent intervention based on a social marketing approach would increase awareness and behavior change among women of reproductive age with SCD. 

Study design

This quasi-experimental study was conducted from 2021 to 2022. Bandar Abbas and Minab, the two largest and most crowded cities of Hormozgan province, Iran, with a high prevalence of SCD, were selected as study settings. This study complies with the Declaration of Helsinki and was performed according to ethics committee approval. The Ethics and Research Committee of the Hormozgan University of Medical Sciences approved the study (approval number: IR.HUMS.REC.1398.486). The records of all patients who provided informed consent for using their data for research purposes were analyzed. In cases of illiteracy, their legal guardians provided informed consent. Statistical analysis was performed with patient anonymity following ethics committee regulations.

Outcome measures

The study's main outcome was the level of engagement in PCHB, which was assessed according to the HBM.

Evaluation instruments

Given that no existing standard questionnaire was available, a questionnaire was developed by the research team based on public databases and the results of other studies [12,13]. A structured questionnaire was used to collect information from participants. A demographic questionnaire, knowledge questionnaire, HBM questionnaire (HBMQ), and PCHB questionnaire were used in this study.

The knowledge questionnaire consisted of 18 multiple-choice questions (e.g., "Preterm birth is common in pregnant women with SCD?"). The answers were "Yes," or "No," or" Do not know." Correct answers received one point each, and not knowing the answer or incorrect answers received no points. Possible scores, therefore, ranged between 0 and 18, with a higher score indicating more awareness.

The HBMQ, a 49-item instrument with six subscales, was designed to assess participants' health beliefs regarding SCD and PCHB. Women's perception of severity related to SCD was measured using the HBMQ severity subscale. This seven-item, five-point Likert-type scale asked the women to indicate whether they perceived SCD as a severe condition (e.g., "Many cases of the birth of a baby with SCD are due to non-participation in preconception care"). The answer choices ranged from "strongly agree" to "strongly disagree." Scale scores ranged from 5 to 35, with higher scores indicating that SCD posed a more significant perceived threat.

Women's perception of risks due to SCD was measured using the HBMQ susceptibility subscale. This 10-item, five-point Likert-type scale asked each woman to think about what she believed to be true about an SCD for her situation (e.g., "Even without obvious symptoms, I can face bad events in pregnancy due to my condition"). The answer choices ranged from "strongly agree" to "strongly disagree." Scale scores ranged from 10 to 50, with higher scores indicating that SCD had a greater perceived risk.

Women's perception of barriers to access to preconception care was measured using an eight-item, 12-point Likert-type scale in the HBMQ. Women were asked to think about what they believed to be accurate about the barriers to preconception care (e.g., "I don't have enough time to participate in preconception visits"). The answer choices for each item ranged from "strongly agree" to "strongly disagree." Scale scores ranged from 12 to 60, with higher scores reflecting an increased perception of barriers to accessing preconception care.

Women's perception of the benefits of preconception care was measured using a seven-item, five-point Likert-type scale in the HBMQ. Women were asked to consider what they believed to be true about the benefits of preconception care (e.g., "Participating in pre-pregnancy care will stop drugs prohibited during pregnancy"). The answer choices for each item ranged from "strongly agree" to "strongly disagree." Scale scores ranged from 7 to 35, with higher scores indicating that preconception care had more significant perceived benefits in preventing the adverse events of SCD.

Women's self-efficacy, a woman's perception of her ability to get preconception care, was measured using a 10-item five-point Likert-type scale in the HBMQ. Women were asked to consider their beliefs, allowing them to get preconception care (i.e., "I can perform the specific tests suggested for preconception counselling"). The answer choices ranged from "strongly agree" to "strongly disagree." Scale scores ranged from 10 to 50, with higher scores reflecting higher self-efficacy for accessing preconception care.

The PCHB was defined as individuals engaging in 12 activities before pregnancy, with each training receiving one point if employed and zero if not. As a result, the PCHB score ranged from 0 to 12. The behaviors included taking 5 mg of folic acid, avoiding smoking, achieving a normal BMI, performing lab tests, vaccination, cervical cytology test, dental visit, avoiding contact with cats, discontinuing hydroxycarbamide three months before conception, visiting a hematologist, genetic screening, and retinal screening.

The demographic factors of each participant, including age, education, occupation of both women and their spouses, and socioeconomic status, were recorded in a simple questionnaire.

A team of nine experts in the fields of health education, epidemiology, obstetrics, and reproductive health was responsible for the validity and reliability of the questionnaire. Minor amendments were made to the wording and order of the questions to achieve a more logical layout. A pilot study was conducted with 30 women to test the items' comprehensibility and establish the questionnaire's reliability. The overall Cronbach's alpha coefficient of the pilot study was calculated to be 0.75 for the maternal knowledge questionnaire and ranged from 0.69 to 0.91 for the HBMQ, indicating that the instrument had a high internal consistency.

Sampling

All women of reproductive age with SCD living in Hormozgan province (the south province of Iran), planning for pregnancy, and who were willing to participate in a study were included. The exclusion criteria were mental disorders, pregnancy during the study period, illiteracy, and not having access to virtual media, such as WhatsApp applications.

Women with SCD were identified using the Iranian integrated health system under the title "SIB." The integrated health system is a national system designed to record, update, and maintain Iranian household health information. This national system records every Iranian family member's vital medical information and disease records. All women of reproductive age with SCD were contacted via telephone and asked if they were planning for pregnancy. Those with positive answers were invited to participate in the study after a comprehensive explanation of the purpose and process of the study; informed consent was taken from the eligible participants. Eligible samples were assigned to the intervention and control groups. We selected eligible participants in Bandar Abbas as controls and those in Minab as intervention groups. It should be noted that all these patients had electronic files in the Iranian integrated health system (SIB system). The reason for selecting Minab as an intervention group was that SCD is more common in Minab than in Bandar Abbas.

The sample size, assuming a minimum participation rate of 40% in two groups, considering an error of 5%, a test power of 90%, and an effect size of 20%, was calculated to be 116 people, considering a dropout of 20%; the final sample size was estimated to be 140 people in each group.

Social marketing-based intervention

Designing the intervention was based on our formative research [13]. The four main components of social marketing were essential in the programs designed to promote preconception care among participants to engage more women in PCHB. According to our formative research, 1) participation in preconception care was set as our "product"; 2) using social media and virtual communication applications, such as WhatsApp, and other traditional promotional channels, such as posters and pamphlets, was our "promotion" strategy; 3) public healthcare centers were selected as a "place" for implementing the intervention; 4) difficult access to medical experts and specialities was the main obstacle to not getting preconception care; therefore, free access to medical experts was set as a "price."

More details on our formative research and the development of an intervention can be accessed in our previously published paper [13].

As a result, the research team, in cooperation with a graphic specialist, created and produced the intervention materials. The central core of the intervention was giving enough information regarding SCD and its adverse events in pregnancy, the benefit of preconception care, the process of preconception care, and empowering women to get preconception care. The research team designed appropriate posters, pamphlets, and videos. These materials were sent to participants in the intervention group via social media. Ten sessions of 20-minute educational classes were also held as another tool to transfer information to participants. To reduce the barriers to accessing preconception care, participants in the intervention group were scheduled for free specialist visits to obstetrician, perinatologist, cardiologist, optometrist, hematologist, and expert in genetic disorders. A reminder was sent to all participants in the intervention group about their appointment with the specialist. The control group received standard routine preconception care offered by healthcare providers in public clinics on demand.

Before implementing the intervention, questionnaires were completed for each participant in both the intervention and control groups. Three months after the end of the intervention, the questionnaires were completed once again for each individual to evaluate the before and after change of information and attitude of individuals.

Statistical analysis

The results were analyzed using IBM SPSS Statistics for Windows, version 25 (released 2017; IBM Corp., Armonk, New York, United States). Descriptive statistics, chi-square test, independent t-test, and paired samples t-test were used to identify and compare the intervention and control groups' demographic information, health beliefs, and PCHB. Analysis of covariance (ANCOVA) was used to compare scores after the intervention, adjusting for pre-intervention scores.

## Results

Comparing the sociodemographic factors of both groups showed significant differences in husbands' education and women's and husbands' employment status, as shown in Table 1.

**Table 1:** Demographic characteristics of the study population SD: Standard deviation. *Data were analyzed using a chi-square test. **Data were analyzed using independent t-test.

Categorical variables	Intervention (n=140)	Control (n=140)		P-value*		
Women education n(%)					0.077	
Primary	9 (6.4)		12 (8.6)			
High-school/diploma	128 (91.5)		120 (85.6)			
Advanced	3 (2.1)		8 (5.8)			
Husband's education n(%)					<0.001	
Primary	13 (9.3)		37 (26.4)			
High-school/diploma	127 (90.7)		76 (54.3)			
Advanced	0		27 (19.3)			
Women's employment status n(%)					<0.001	
Housewife	109 (77.9)		93 (66.4)			
Employee	31 (22.1)		47 (33.6)			
Husband's employment status n(%)					<0.01	
No job	15 (10.7)		20 (14.3)			
Employee	125 (89.3)		120 (85.7)			
Household income n(%)					0.533	
Poor	26 (18.6)		21 (15.0)			
Average	75 (53.6)		84 (60.0)			
Good	39 (27.9)		35 (25.0)			
Continuous variables (mean ± SD)	Intervention		Control		P-value**	
Woman's age	27.14±4.15		27.15±4.61		0.978	
Husband's age	30.34±4.64		29.94±4.72		0.457	

The difference in the mean scores of awareness and constructs of HBM in the intervention and control groups at baseline and after the intervention was investigated using an independent t-test. According to the analysis, self-efficacy was the only factor that was not significantly different between the two groups at baseline. This means that the two groups were heterogeneous regarding awareness, perceived severity, perceived susceptibility, perceived benefits, and perceived barriers. On the other hand, after the intervention, those in the intervention group had higher scores in perceived severity, perceived susceptibility, perceived benefits, and self-efficacy. The control group had more heightened awareness and perceived barrier scores (Table 2). In the present study, the paired t-test results showed that awareness, perceived severity, perceived susceptibility, perceived benefits, and self-efficacy increased in both groups while perceived barriers decreased (Table 2).

**Table 2:** Comparison of the awareness and health belief model constructs in the baseline and after intervention in the two research groups SD: Standard deviation. *Data were analyzed using a paired t-test. **Data were analyzed using independent t-test.

Constructs (mean ± SD)	Intervention	Control	P-value**
Awareness			
Baseline	16.31±2.63	14.95±1.32	<0.001
After intervention	17.67±0.47	18.15±1.69	<0.001
*P-value	<0.001	<0.001	
Perceived severity			
Baseline	20.49±3.34	19.55±3.32	<0.01
After intervention	33.84±2.76	28.22±3.78	<0.001
*P-value	<0.001	<0.001	
Perceived susceptibility			
Baseline	25.21±6.82	31.79±4.28	<0.001
After intervention	49.05±1.06	32.84±4.52	<0.001
*P-value	<0.001	<0.001	
Perceived benefits			
Baseline	18.92±2.68	20.35±4.15	<0.01
After intervention	33.58±1.88	31.62±2.64	<0.001
*P-value	<0.001	<0.001	
Perceived barriers			
Baseline	41.61±6.45	40.67±4.91	<0.001
After intervention	16.98±2.80	35.68±5.96	<0.001
*P-value	<0.001	<0.001	
Self-efficacy			
Baseline	33.34±4.15	33.37±4.03	0.953
After intervention	46.52±3.51	43.88±6.30	<0.001
*P-value	<0.001	<0.001	

**Figure 1:**
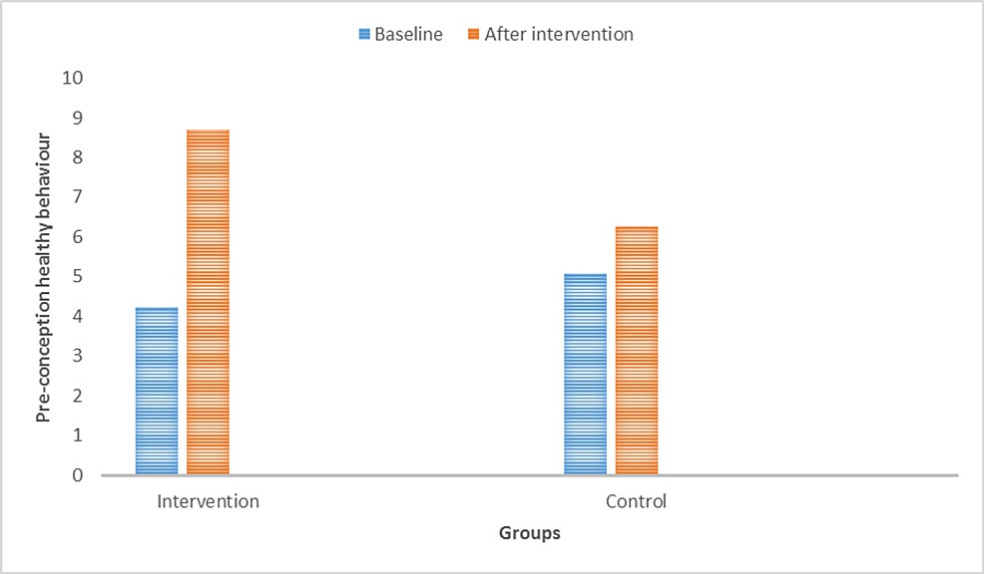
Comparison of the preconception healthy behavior scores at baseline and after intervention in each group Based on paired t-test.

We compared the PCHB scores of both groups. As shown in Figure 1, employing healthy behavior was more dominant in the intervention group.

ANCOVA was performed to control and moderate the effect of scores in the pretest and the demographic factors (husbands' education and women's and husbands' employment status). As shown in Table 3, between the adjusted averages, awareness scores (F = 526.81, p < 0.001), perceived susceptibility (F = 2000.98, p < 0.001), perceived severity (F = 195.66, p < 0.001), perceived benefits (F = 70.93, p > 0.001), perceived barriers (F = 1214.41, p > 0.001), self-efficacy (F = 18.78, p > 0.001), and engagement in the PCHB (F = 15.44, p < 0.001) were significantly different between the control and intervention groups after the intervention. Therefore, the results indicate the positive effect of the social marketing-based intervention on the constructs of the HBM and the level of engagement in PCHB in the intervention group participants.

**Table 3:** Analysis of covariance to adjust the pretest scores as a variable covariate PCHB: preconception healthy behavior, df: degree of freedom

Variables	Source	Sum of squares	df	Mean square	F-value	P-value
Awareness	Group	380.67	1	380.67	526.81	<0.001
Perceived susceptibility	Group	1699.902	1	1699.902	200.980	<0.001
Perceived severity	Group	1955.47	1	1955.47	195.66	<0.001
Perceived benefits	Group	338.98	1	338.98	70.93	<0.001
Perceived barriers	Group	2482.046	1	2482.046	121.414	<0.001
Self-efficacy	Group	489.02	1	489.02	18.78	<0.001
PCHB	Group	450.37	1	450.37	15.44	<0.001

A multivariate linear regression analysis was used to test the effect of awareness and each construct's HBM on PCHB. The dependent variable was PCHB, and awareness and other constructs were the independent variables. As shown in Table 4, awareness, perceived susceptibility, and self-efficacy were the strong predictors of PCHB.

**Table 4:** Predictors of PCHB in the intervention group based on the HBM constructs PCHB: preconception healthy behavior, HBM: health belief model

Variables	B	95.0% confidence Interval	Standardized coefficients	t	P-value
Awareness	0.198	0.085	0.192	2.249	0.026
Perceived susceptibility	0.065	0.026	0.248	2.447	0.016
Perceived severity	0.012	0.034	0.033	0.368	0.714
Perceived benefits	0.001	0.049	0.002	0.025	0.980
Perceived barriers	-0.025	0.022	-0.105	-1.146	0.254
Self-efficacy	0.086	0.026	0.264	3.270	<0.001

## Discussion

According to our findings, a social marketing-based intervention raised participants' awareness of SCD and the importance of preconception care. This was also seen in the control group, which received standard care; surprisingly, it was even higher than in the intervention group. This might be due to the different sources from which individuals can access information. As we mentioned in the methods, those in the control group also received standard care, a routine visit to healthcare providers; therefore, we assume they might get their information this way. Even though the control group were more knowledgeable, their engagement in PCHB was lower than in the intervention group. This emphasizes the significance of other determinants in health behavior. According to the HBM construct analysis, the intervention group perceived SCD as a high-risk condition compared to the control group and at greater risk after the intervention. They also believed that preconception care could help them avoid adverse events and feel more capable of engaging in preconception activities. They also perceived fewer barriers to obtaining preconception care.

The review of intervention studies based on the social marketing approach shows that the success of such an approach was through increasing awareness [14]; changing, improving, and modifying attitudes [15]; and changing behavior [16]. Many examples of successful interventions based on social marketing in areas, such as the use of nutritional supplements among women of reproductive age [17], encouraging women to participate more in prenatal care [18], promoting natural childbirth [19], promotion of COVID-19 vaccination [20], smoking cessation [21], AIDS control [22], and healthy eating [23], have been reported.

The main purpose of these studies has been to change behaviors (both undesirable and desired behaviors) [24].

In all the studies mentioned above, the change in the behavior of the study participants has been interpreted with the help of health behavior theories. For example, in the study by Darsarah et al., who promoted natural childbirth through a social marketing campaign, the desire of more people to choose natural birth was created by increasing people's awareness and self-efficacy [19]. Another social marketing campaign entitled "Ante La Duda, Pregunta" (translated as "When in doubt, ask") in Puerto Rico was successful in raising awareness about the full range of reversible contraceptive methods [25]. 

Our findings showed significant differences in awareness and constructs of the HBM (perceived sensitivity, perceived severity, self-efficacy, perceived benefits, and perceived barriers) following intervention. In our research, awareness, perceived susceptibility, and self-efficacy strongly predicted women's engagement in PCHB. Our intervention raised public awareness of preconception care and encouraged women with SCD to engage more in PCHB. This success is due to the use of a social marketing approach. Knowing the target population, their health needs, and priorities and using people's opinions to remove the obstacles and create the necessary facilities for adopting healthy behavior is one of the most important goals of social marketing, which is well evident in the research based on this approach.

Not all social marketing interventions were successful, according to the literature. Among the unsuccessful examples of interventions based on social marketing in changing behavior, we can mention a study to investigate the effect of social marketing-based intervention in preventing syphilis in gay men. In this study, there was no change in the number of clinic visits for early disease diagnosis or receiving medical services following the implementation of social marketing-based interventions. In general, in this research, the desired goals of the program were not achieved. The designers of this intervention believed that the main reason for the failure of this intervention was the use of an inappropriate communication channel because only 36.5% of the people in the intervention group had encountered the tools used, such as posters, banners, and pamphlets [26].

Generally, in social marketing, by examining society's health problems and their prioritization, the health problem is identified as a priority, and the behavioral factors affecting it are known. Then, the work's main framework is determined by determining the general objectives of the program, organizing the preliminary agenda for evaluation, and estimating the costs needed for the program's design and implementation [27]. Knowing the audience, formulating the appropriate content of the health message, and using the proper communication methods with the audience obtained from the formative research are among the most critical success principles of an intervention based on social marketing [28].

One of the strengths of the current research was the simultaneous use of HBM and the concepts of social marketing and the combination of the two to make the intervention more effective on the audience. Moreover, conducting formative research to know the audience and design an intervention based on the principles of social marketing is one of the highlights of this study. However, this study, like any other study, has limitations. One of these limitations is the failure to evaluate the process. One of the most important steps in monitoring and evaluating interventions based on social marketing is to assess three levels: formative, process, and cumulative. In the current study, formative evaluation was done through qualitative and quantitative. A cumulative evaluation was also done by comparing knowledge and attitude and the level of people's participation in preconception care. However, the evaluation of the process, which examines the methods and tasks related to implementing the intervention programs and shows us how much the intervention programs have received the audience's attention, was not done. Since this study was conducted mainly during the COVID-19 pandemic, it is possible that the interventions and the details of the activities were not done according to what was designed, which can affect the study results.

## Conclusions

Social marketing-based intervention successfully increased the awareness and enhanced the health beliefs of women with SCD regarding preconception care. This intervention also increased PCHB among women of reproductive age with SCD. It is critical to use health education models to promote preventive behaviors as a theoretical framework for interventions to reduce maternal and fetal complications of SCD. A social marketing approach is recommended to promote desirable healthy behaviors and prevent unhealthy behaviors.

## References

[1] Aghamolaei T, Pormehr-Yabandeh A, Hosseini Z, Roozbeh N, Arian M, Ghanbarnezhad A (2022). Pregnancy in the sickle cell disease and fetomaternal outcomes in different sickle cell genotypes: a systematic review and meta-analysis. Ethiop J Health Sci.

[2] Sundd P, Gladwin MT, Novelli EM (2019). Pathophysiology of sickle cell disease. Annu Rev Pathol.

[3] Posner SF, Johnson K, Parker C, Atrash H, Biermann J (2006). The national summit on preconception care: a summary of concepts and recommendations. Matern Child Health J.

[4] McDougall B, Kavanagh K, Stephenson J, Poston L, Flynn AC, White SL (2021). Health behaviours in 131,182 UK women planning pregnancy. BMC Pregnancy Childbirth.

[5] Movahed E, Rezaee Moradali M, Saeed Jadgal M, Zareipour M, Tasouji Azari M (2022). Effectiveness of the application of an educational program based on the Health Belief Model (HBM) in Adopting. Invest Educ Enferm.

[6] Khekade H, Potdukhe A, Taksande AB, Wanjari MB, Yelne S (2023). Preconception care: a strategic intervention for the prevention of neonatal and birth disorders. Cureus.

[7] Teshome F, Kebede Y, Abamecha F, Birhanu Z (2020). Why do women not prepare for pregnancy? Exploring women's and health care providers' views on barriers to uptake of preconception care in Mana District, Southwest Ethiopia: a qualitative study. BMC Pregnancy Childbirth.

[8] Akbar MB, Foote L, Lawson A, French J, Deshpande S, Lee NR (2021). The social marketing paradox: challenges and opportunities for the discipline.

[9] Girma A, Bedada A, Kumbi S (2023). Utilization of preconception care and associated factors among pregnant women attending ANC in private MCH Hospitals in Addis Ababa, Ethiopia. BMC Pregnancy Childbirth.

[10] Suto M, Mitsunaga H, Honda Y, Maeda E, Ota E, Arata N (2021). Development of a health literacy scale for preconception care: a study of the reproductive age population in Japan. BMC Public Health.

[11] Prue CE, Daniel KL (2006). Social marketing: planning before conceiving preconception care. Matern Child Health J.

[12] Darsareh F, Aghamolaei T, Rajaei M, Madani A, Zare S (2016). The differences between pregnant women who request elective caesarean and those who plan for vaginal birth based on Health Belief Model. Women Birth.

[13] Pormehr-Yabandeh A, Aghamolaei T, Hosseini Z, Roozbeh N, Ghanbarnezhad A (2022). Preconception care counselling among women with sickle cell anaemia in the south of Iran: a qualitative study based on social marketing model. J Obstet Gynaecol.

[14] Plant A, Montoya JA, Rotblatt H (2010). Stop the sores: the making and evaluation of a successful social marketing campaign. Health Promot Pract.

[15] Huhman M, Kelly RP, Edgar T (2017). Social marketing as a framework for youth physical activity initiatives: a 10-year retrospective on the legacy of CDC's VERB Campaign. Curr Obes Rep.

[16] Campbell MA, Finlay S, Lucas K, Neal N, Williams R (2014). Kick the habit: a social marketing campaign by Aboriginal communities in NSW. Aust J Prim Health.

[17] Warnick E, Dearden K, Slater S, Butrón B, Lanata C, Huffman S (2004). Social marketing improved the use of multivitamin and mineral supplements among resource-poor women in Bolivia. J Nutr Educ Behav.

[18] Vonderheid SC, Carrie SK, Norr KF, Grady MA, Westdahl CM (2013). Using focus groups and social marketing to strengthen promotion of group prenatal care. ANS Adv Nurs Sci.

[19] Darsareh F, Aghamolaei T, Rajaei M, Madani A, Zare S (2019). B Butterfly Campaign: a social marketing campaign to promote normal childbirth among first-time pregnant women. Women Birth.

[20] Bardus M, Assaf SA, Sakr CJ (2023). Using social marketing to promote COVID-19 vaccination uptake: a case study from the "AUBe Vaccinated" Campaign. Vaccines (Basel).

[21] Hastings G, McLean N (2006). Social marketing, smoking cessation and inequalities. Addiction.

[22] Sewak A, Singh G (2017). Integrating social marketing into Fijian HIV/AIDS prevention programs: lessons from systematic review. Health Commun.

[23] Nosi C, D'Agostino A, Pratesi CA, Barbarossa C (2021). Evaluating a social marketing campaign on healthy nutrition and lifestyle among primary-school children: A mixed-method research design. Eval Program Plann.

[24] Shams M, Shojaeizadeh D, Majdzadeh R, Rashidian A, Montazeri A (2011). Taxi drivers' views on risky driving behavior in Tehran: a qualitative study using a social marketing approach. Accid Anal Prev.

[25] Powell R, Rosenthal J, August EM (2022). Ante La Duda, Pregunta: a social marketing campaign to improve contraceptive access during a public health emergency. Health Commun.

[26] Darrow WW, Biersteker S (2008). Short-term impact evaluation of a social marketing campaign to prevent syphilis among men who have sex with men. Am J Public Health.

[27] Morris ZS, Clarkson PJ (2009). Does social marketing provide a framework for changing healthcare practice?. Health Policy.

[28] Gordon R, McDermott L, Stead M, Angus K (2006). The effectiveness of social marketing interventions for health improvement: what's the evidence?. Public Health.

